# Proteasome Activity Profiling Uncovers Alteration of Catalytic β2 and β5 Subunits of the Stress-Induced Proteasome during Salinity Stress in Tomato Roots

**DOI:** 10.3389/fpls.2017.00107

**Published:** 2017-02-03

**Authors:** Judit Kovács, Péter Poór, Farnusch Kaschani, Balakumaran Chandrasekar, Tram N. Hong, Johana C. Misas-Villamil, Bo T. Xin, Markus Kaiser, Herman S. Overkleeft, Irma Tari, Renier A. L. van der Hoorn

**Affiliations:** ^1^Department of Plant Biology, University of SzegedSzeged, Hungary; ^2^Chemical Biology, Fakultät für Biologie, Zentrum für Medizinische Biotechnologie, Universität Duisburg-EssenEssen, Germany; ^3^Plant Chemetics Laboratory, Department of Plant Sciences, University of OxfordOxford, UK; ^4^Plant Chemetics Laboratory, Max Planck Institute for Plant Breeding ResearchCologne, Germany; ^5^Botanical Institute and Cluster of Excellence on Plant Sciences, University of CologneCologne, Germany; ^6^Leiden Institute of Chemistry, Leiden UniversityLeiden, Netherlands

**Keywords:** 20S proteasome, immune proteasome, activity-based protein profiling, programmed cell death, salt stress, tomato root, catalytic subunit

## Abstract

The stress proteasome in the animal kingdom facilitates faster conversion of oxidized proteins during stress conditions by incorporating different catalytic β subunits. Plants deal with similar kind of stresses and also carry multiple paralogous genes encoding for each of the three catalytic β subunits. Here, we investigated the existence of stress proteasomes upon abiotic stress (salt stress) in tomato roots. In contrast to *Arabidopsis thaliana*, tomato has a simplified proteasome gene set with single genes encoding each β subunit except for two genes encoding β2. Using proteasome activity profiling on tomato roots during salt stress, we discovered a transient modification of the catalytic subunits of the proteasome coinciding with a loss of cell viability. This stress-induced active proteasome disappears at later time points and coincides with the need to degrade oxidized proteins during salt stress. Subunit-selective proteasome probes and MS analysis of fluorescent 2D gels demonstrated that the detected stress-induced proteasome is not caused by an altered composition of subunits in active proteasomes, but involves an increased molecular weight of both labeled β2 and β5 subunits, and an additional acidic pI shift for labeled β5, whilst labeled β1 remains mostly unchanged. Treatment with phosphatase or glycosidases did not affect the migration pattern. This stress-induced proteasome may play an important role in PCD during abiotic stress.

## Introduction

The proteasome plays a key role in protein degradation of cytonuclear proteins during biotic and biotic stress in plants. The 26S proteasome is a highly conserved protein complex which has a crucial role in selective protein degradation during cell death and development. The 26S proteasome consists of a catalytic 20S core protease and a 19S regulatory particle (Coux, 1996; Kurepa and Smalle, 2008). The core protease is composed of four heptameric rings; two outer α-rings and two inner β-rings, each of them composed of seven different α- and β subunits (Löwe et al., 1995). These four heptameric rings are arranged as a cylinder with three large internal chambers. The active sites are in the central chamber, residing in three catalytic subunits: β1, β2, and β5 which cleave after acidic, basic, and hydrophobic residues and representing caspase-like, trypsin-like, and chymotrypsin-like activities, respectively (Löwe et al., 1995; Kurepa and Smalle, 2008; Murata et al., 2009).

In addition to the standard β1, β2, and β5 subunits, animals have additional “immuno”-subunits (β1i, β2i, and β5i) and a thymus-specific subunit (β5t; Klare et al., 2007; Murata et al., 2007). Replacement of standard catalytic subunits by the catalytic “immuno”-subunits creates the immune proteasome (i-20S; Aki et al., 1994). These “immuno”-subunits can also co-exist with standard subunits within the same proteasome complex, creating an intermediate-type proteasome (Klare et al., 2007). Intermediate- and immune proteasomes play roles in the degradation of damaged and misfolded proteins during cell death and disease in animals (Zheng et al., 2012; Grigoreva et al., 2015).

Interestingly, there are indications of the existence of an alternative proteasome in plants. A defense-induced β1 subunit (β1din) in tobacco suggests the presence of a “defense proteasome” in plants (Suty et al., 2003). Transcripts of β*1din* accumulate during the elicitin-induced response in tobacco (Lequeu et al., 2005) whereas salicylic acid signaling activates the proteasome post-translationally (Gu et al., 2010). But little is known about modification and activation of specific proteasome subunits in plants. An optimal 26S proteasome is essential for maintaining plant drought stress tolerance (Cho et al., 2008; Yee and Goring, 2009). For instance, regulatory particle mutants have increased oxidative stress tolerance (Kurepa et al., 2008), and *rpn1a* mutants have increased salt hypersensitivity (Wang et al., 2009).

In this study, we investigated the proteasome during abiotic stress. We focused our studies on salt stress-induced PCD in tomato roots. The root is the primary organ recognizing salt stress and initiating signaling pathways. A moderate salt concentration stops root growth but higher salt concentrations can induce PCD, characterized by an oxidative burst, cytochrome c release, and DNA fragmentation (Giannattasio et al., 2008; Shabala, 2009; Andronis and Roubelakis-Angelakis, 2010), all triggered by sodium ions entering the cell (Shabala, 2000; Zhu, 2001). PCD in root tips upon salt stress is thought to be a defensive response during development to maintain the integrity of the root system (Hasegawa et al., 2000; Huh et al., 2002; Gémes et al., 2011). However, when lethal salt exposure is prolonged, it leads to death not only at the cellular level but also at tissue or organ level (Bagniewska-Zadworna and Arasimowicz-Jelonek, 2016).

Here, we tested the hypothesis that also plants have a stress-induced proteasome. We used ABPP (Cravatt et al., 2008) and proteomics on salt-stress in tomato roots to investigate the molecular composition of this stress-induced proteasome. ABPP involves biotinylated or fluorescent chemical probes that react with the active site of enzymes in an activity-dependent manner, creating an irreversible covalent bond that facilitates detection and identification (Morimoto and Van der Hoorn, 2016). ABPP displays changes in the activity level of the enzymes upon different treatments, for instance in the activity of PLCPs and serine hydrolases upon biotic stress, or vacuolar processing enzymes and proteasome during PCD (Shabab et al., 2008; Kaschani et al., 2009; Gu et al., 2012; van der Linde et al., 2012; Misas-Villamil et al., 2013a,b; Sueldo et al., 2014). Our ABPP studies reveal previously unknown stress-associated modifications of the proteasome in tomato roots upon salt stress.

## Materials and Methods

### Bioinformatics

Genes encoding the β subunits of tomato were identified by BLASTp searches of the predicted proteome (ITAG release 2.40) for homologs of the seven Arabidopsis β subunits at the SolGenomics website^1^. The β2a protein sequence was modeled onto polypeptide H of the structure of the yeast proteasome (2zcy, Groll et al., 2008) using Swiss Model^2^ (Biasini et al., 2014). This β2a model was used in PyMol to replace the β2 in the structure of the yeast proteasome. Only the surface of one ring of β subunits was visualized and the various parts and residues were colored using PyMol. Further annotations were added using CorelDRAW. Transcript levels were extracted from published RNA sequencing experiments on different organs (The Tomato Genome Consortium, 2012).

### Plant Materials and Growth Conditions

Tomato (*Solanum lycopersicum* L. cv “Rio Fuego”) plants were germinated at 26°C for 3 days in the dark, and the seedlings were subsequently transferred to perlite for 2 weeks. Plants were grown hydroponically in a controlled environment in a greenhouse (300 μmol m^-2^ s^-1^ photon flux density with 12/12 light/dark photoperiod, 25°C, and 55–60% relative humidity) for 3 weeks (Poór et al., 2011). Tomato plants were treated with 0-, 100-, and 250 mM NaCl in the nutrient solution [2 mM Ca(NO_3_)_2_, 1 mM MgSO_4_, 0.5 mM KCl, 0.5 mM, KH_2_PO_4_, 0.5 mM Na_2_HPO_4_, 0.001 mM MnSO_4_, 0.005 mM ZnSO_4_, 0.0001 mM (NH_4_)_6_Mo_7_O_24_, 0.01 mM H_3_BO_4_, 0.02 mM Fe(III)-EDTA]. Samples were made in at 9 a.m. and samples were taken in triplicate at 1, 6, and 24 h after salt exposure.

### FDA Staining

Fluorescein diacetate (FDA; Sigma–Aldrich, St. Louis, MO, USA) was used to determine cell viability according to Gémes et al. (2011). Root tip segments were stained for 10 min at room temperature in the dark with 10 mM FDA dissolved in 3 ml 10 mM 2-(*N*-morpholino)ethanesulfonic acid (MES) potassium chloride (KCl) buffer (pH 6.15). After staining, the samples were washed two times in 10 min with MES/KCl buffer (pH 6.15). Fluorescence intensity was detected with Zeiss Axiovert 200 M type fluorescent microscope (Carl Zeiss Inc., Jena, Germany) equipped with an 5X objective. Digital photographs were taken from the samples with a high-resolution digital camera (Axiocam HR, HQ CCD camera; Carl Zeiss Inc., Jena, Germany) using a filter set 10 (excitation 450–495 nm, emission 515–565 nm) or filter set 20HE (excitation: 535–585 nm, emission: 600–655 nm). The fluorescence emission (pixel intensity) was measured on digital images with AXIOVISION REL. 4.8 software (Carl Zeiss Inc., Munich, Germany).

### Small-Scale Labeling Reaction

#### Sample Preparation

Root tissue was homogenized in 50 mM Tris buffer at pH 7.5 containing 5 mM DTT for labeling of the proteasome. The extract was mixed and centrifuged at 10000 g for 10 min at 4°C to remove cell debris and the supernatant was collected and used for labeling.

#### Labeling of Proteasome Subunits

Hundred microgram/milliliterprotein extract was labeled with 2 μM MV151 for 3 h or 0.2 μM MVB072 or co-labeled with 0.8 μM LW124/MVB127 for 2 h at room temperature in the dark in 60 μl total volume. Equal volumes of DMSO were added for the no-probe-control. For inhibition assays, extracts were pre-incubated with 50 or 100 μM epoxomicin or 50 μM N3β1 or N3β5 or DMSO and these extracts were labeled with the suitable probe. The labeling reactions were stopped by adding gel loading buffer containing β-mercaptoethanol at 4X final concentration and heating at 95°C for 10 min. The reaction mixture was separated on 15% SDS gel at 200 V for 75 min. Labeled proteins were visualized by in-gel fluorescence scanning using a Typhoon 9400 Imager (GE Healthcare)^3^ using excitation and emission wavelengths of 532/580 nm for MV151, MVB072, and MVB127 and of 470/530 nm for LW124. 532/580 nm and 470/530 were overlaid and signals were quantified using ImageJ 1.48V.

### IEF 2D SDS PAGE

Labeled and precipitated proteins were resuspended in UTC buffer (8 M urea, 2 M thiourea, 4% (w/v) CHAPS, 1 g AG 501-X8 Resin) containing 1% (v/v) ampholyte and 65 mM DTT. Samples were isoelectrically focused on 7 cm immobilized pH gradient (IPG) 3–10 pH strips (BioRad–ReadyStrip^TM^ IPG Strips) using BioRad PROTEAN i12 IEF system with the following focusing conditions: 12 h passive rehydration; 250 V, 15 min, rapid ramp; 4000 V, 1 h, slow ramp; 4000 V, 30000 Vhr, rapid ramp; 500 V hold. After focusing, IPG strips were equilibrated in IEF Equilibration buffer [6 M urea, 5% SDS (w/v), 30% glycerol (v/v)] containing 1 % (w/v) DTT, then in IEF Equilibration buffer containing 2.5% iodoacetamide (w/v). The second dimension electrophoresis was run on a 15% SDS gel. Gels were imaged using a Typhoon 9400 Imager (GE Healthcare) using excitation and emission wavelengths of 532/580 nm. Images were quantified using ImageJ 1.48V by multiplication of the fluorescence intensity and the area of each of the spots (*n* = 3).

### In-Gel Digestion and MS

Bands were excised by hand and treated with trypsin as described elsewhere (Shevchenko et al., 2006). Tryptic digests were desalted on home-made C18 StageTips as described (Rappsilber et al., 2007). After elution from the StageTips samples were dried using a vacuum concentrator (Eppendorf) and the peptides taken up in 10 μL 0.1 % formic acid solution. LC-MS/MS experiments were performed on an Orbitrap Elite instrument (Thermo, Michalski et al., 2012) that was coupled to an EASY-nLC 1000 liquid chromatography (LC) system (Thermo). The LC was operated in the two-column mode. The home-made fused silica column equipped with a glass fiber frit (Maiolica et al., 2005) was packed with Reprosil-Pur 120 C18-AQ 3 μm resin (Dr. Maisch) and connected to the analytical column via an UHPLC union (Upchurch; UH-432). The analytical column was a fused silica capillary (75 μm × 25 cm) with integrated PicoFrit emitter (New Objective) packed in-house with Reprosil-Pur 120 C18-AQ 3 μm resin (Dr. Maisch). The analytical column was attached to a nanospray flex ion source (Thermo). The LC was equipped with two mobile phases: solvent A (0.1% formic acid, FA, in UPLC grade water) and solvent B (0.1% FA in acetonitrile, ACN). Peptides were delivered to the pre-column via the integrated autosampler at a flow rate of 2–3 μl/min in 100% solvent A. Peptides were subsequently separated on the analytical column by running a 70 min gradient of solvents A and B (start with 7% B; gradient 7–35% B for 60 min; gradient 35–100% B for 5 min and 100% B for 5 min) at a flow rate of 300 nl/min. The mass spectrometer was operated using Xcalibur software (version 2.2 SP1.48) and was set in the positive ion mode. Precursor ion scanning was performed in the Orbitrap analyzer (FTMS) in the scan range of m/z 300–1,500 and at a resolution of 120,000 with the internal lock mass option turned on (lock mass was 445.120025 m/z, polysiloxane; Olsen et al., 2005). Product ion spectra were recorded in a data dependent fashion in the ion trap (ITMS) in a variable scan range and at a rapid scan rate. The ionization potential (spray voltage) was set to 1.6–2.0 kV. Peptides were analyzed using a repeating cycle consisting of a full precursor ion scan (1.0 × 10^6^ ions) followed by 15 product ion scans (1.0 × 10^4^ ions) where peptides are isolated based on their intensity in the full survey scan (threshold of 500 counts) for tandem mass spectrum (MS2) generation that permits peptide sequencing and identification. CID collision energy was set to 35% for the generation of MS2 spectra. During MS2 data acquisition dynamic ion exclusion was set to 120 s with a maximum list of excluded ions consisting of 500 members and a repeat count of one. Ion injection time prediction, preview mode for the FTMS, monoisotopic precursor selection and charge state screening were enabled. Only charge states bigger than 1 were considered for fragmentation.

### Peptide and Protein Identification

The recorded RAW files were processed in ProteomeDiscoverer 1.4 (PD14, Thermo). MS2 spectra were extracted using the Spectrum Selector node. Precursor selection was set to “use MS1 precursor.” The mass range was set between 350 and 5,000 Da with a minimum peak count of 1. Mass analyzer was set to “any” and MS order to “MS2.” Activation type was set to “is CID” and Scan type was defined as “full” with ionization source set to “is nanospray.” Selected spectra were submitted to the in house MASCOT server [version 2.4.1 (Perkins et al., 1999)] using the PD14 MASCOT node.

Tandem mass spectrum spectra data were searched against the tomato_ITAG.fasta database^4^ (version 2.3; 34725 entries). All searches included a contaminants database (as implemented in MASCOT and MaxQuant, 263 sequences). The contaminants database contains known MS contaminants and was included to estimate the level of contamination. Mascot and Andromeda searches allowed for oxidation of methionine residues (16 Da) and a static modification on cysteine (57 Da, alkylation with iodoacetamide). Enzyme specificity was set to Trypsin/P. The instrument type in MASCOT searches was set to ESI-TRAP and the mass tolerance was set to ± 10 ppm for precursor mass and ± 0.35 Da for product ion masses. MS2 spectra matches were then evaluated using the peptide validation node of PD14 with the standard settings [search against decoy database, target false discovery rate (FDR, strict): 0.01 and target FDR (released): 0.05]. The reported results were further filtered. On peptide level only peptides with a minimum confidence ‘medium’ were reported and on protein level only proteins with a minimum of at least two peptide hits were reported.

### Protein Phosphatase Treatment

Sixty microliter of MVB072-labeled sample containing 1 M NaCl, 25 mM MgCl_2_ 10 mM DTT, and 12.5X protease inhibitor cocktail (Sigma) were treated with 1 or 5 μl of alkaline phosphatase (Sigma P0114) and incubated at 37°C for 1 h. Samples were analyzed on 16% SDS-PAGE, whereas the remainder of the sample were separated on 10% SDS-PAGE and transferred to polyvinylidene difluoride (PVDF) membrane for detection of phosphorylated MAPK using primary antibody, Phospho-p44/42 MAPK (Erk1/2) (Thr202/Tyr204) antibody (CST, #9101) and secondary Goat anti-Rabbit IgG (Thermo, #31466) and visualized using chemiluminescent substrates (SuperSignal West/Pico Chemiluminescent substrates, Thermo scientific).

### PNGaseF Treatment of Labeled Proteins

Nine microliter of MVB072-labelled tomato root extract and Bovine Fetuin (Promega) were treated with 1 μl of 10X glycoprotein denaturing buffer (New England BioLabs) and heated at 95°C for 5 min. The denatured proteins were chilled on ice. Two microliter 10X GlycoBuffer (New England BioLabs), 2 μl 10% NP40 (Promega), and 6 μl H_2_O was added to the reaction. The mixture was treated with 1 μl PNGase F (New England BioLabs) or with 1 μl H_2_O and incubated at 37°C for 1 h. Samples were analyzed on 16% SDS-PAGE.

### Protein Deglycosylation of Labeled Proteins

Eighteen microliter of MVB072-labelled sample and Bovine Fetuin (Promega) were treated with 2 μl of 10X denaturing solution (Promega) and heated at 95°C for 10 min. The denatured proteins were chilled on ice for 5 min. To the denatured samples 5 μl of 10X Deglycosylation Reaction Buffer (Promega), 5 μl of 10% NP40 (Promega), and 15 μl of water were added. Samples were treated with 5 μl of Protein Deglycosylation Mix (Promega – PNGase F, *O*-Glycosidase, Neuraminidase, β1–4 Galactosidase, β-*N*-Acetylglucosaminidase) and incubated at 37°C for 8 h. Samples were analyzed on 16% SDS-PAGE.

## Results

### Tomato Has Eight Genes Encoding the Seven β Subunits

**FIGURE 1:**
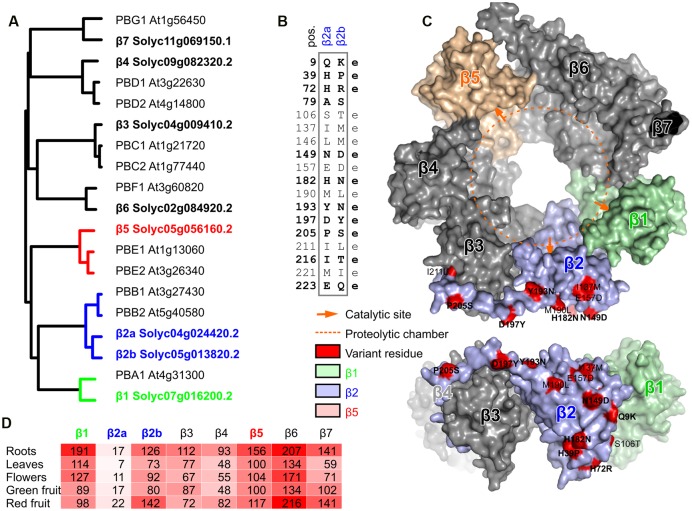
**Phylogeny and variation of beta proteasome subunits of tomato. (A)** Phylogenetic tree of beta subunit genes of tomato and Arabidopsis. Neighbor-joining tree of protein sequences was build using ClustalW2. **(B)** Summary of the variant amino acid residues that differ between β2a and β2b. e, putative solvent-exposed. Significant variation is printed in bold. **(C)** Location of variant residues in β2, modeled on the yeast proteasome. The tomato β2a protein was modeled using the β2 of yeast (2zcy) as a template. Residues that differ between β2a and β2b are highlighted in red in the topview (top) and sideview (bottom) of the β-ring of the proteasome and summarized in the table. The proteolytic chamber is highlighted with a dashed orange line and catalytic sites are indicated with orange arrows. **(D)** Transcript levels of β subunit-encoding genes in various tomato organs. These data were extracted from The Tomato Genome Consortium (2012). Reads per kilobase of transcripts per million mapped reads (RPKM) values were extracted from the database for each gene.

To investigate the tomato proteasome, we performed BLAST searches with the Arabidopsis β subunits on the predicted tomato proteome^5^ and identified eight tomato genes encoding β subunits. Phylogenetic analysis of the tomato and Arabidopsis β subunits revealed that tomato genome has one gene for each of the six β subunits (β1, β3, β4, β5, β6, and β7), and two genes encoding β2 (**Figure 1A**). The two β2 proteins in tomato (β2a and β2b) are more closely related to each other when compared to the two β2 proteins of Arabidopsis (PBB1 and PBB2), consistent with the fact that genome duplication occurred in each lineage, after divergence (The Tomato Genome Consortium, 2012). Tomato, however, must have lost the paralogous copies of each except one proteasome β subunit. Sequence alignment with the Arabidopsis orthologs indicates that each of the eight tomato genes encodes a putative functional subunit, including an N-terminal pro-domain for all subunits and a catalytic Thr for the β1, β2, and β5 subunits (Supplementary Figure S1).

The branch lengths indicate that the two β2 subunits are substantially different (**Figure 1A**). Indeed, we counted 18 amino acid residues that differ between the mature β2a and β2b subunits (**Figure 1B**). Most of these amino acid substitutions are biochemically dissimilar. To estimate if these variant residues can affect the proteolytic chamber, we generated a structural model of the tomato β2 protein using the yeast proteasome (2zcy, Groll et al., 2008) as a template. Mapping the residues that vary between β2a and β2b onto the structural model revealed that none of the variant residues are exposed to the proteolytic chamber (**Figure 1C**). Interestingly, nearly all the variant residues reside on the outer surface and are likely solvent-exposed (**Figure 1C**).

To determine which of the β subunit-encoding genes are expressed in different tissues, we mined RNAseq datasets for the transcript levels of each of these genes from RNAseq data (The Tomato Genome Consortium, 2012). As expected for subunits that assemble in stoichiometric complexes, transcript levels of each of the β subunit genes are very similar, with the exception of β*2a* transcripts, which accumulate 5- to 10-fold lower when compared to β*2b* and the other β subunit-encoding transcripts (**Figure 1D**). Nevertheless, detection of β*2a* transcripts suggests that β*2a* is not a pseudogene, but the transcript levels are low under normal conditions. The ratio between β*2a* and β*2b* transcript levels does not significantly change in different tissues (**Figure 1D**), suggesting that proteasome assembly might be similar for β2a and β2b subunits in different tissues.

### Salt Treatment Induces Loss of Viability in Tomato Roots

**FIGURE 2:**
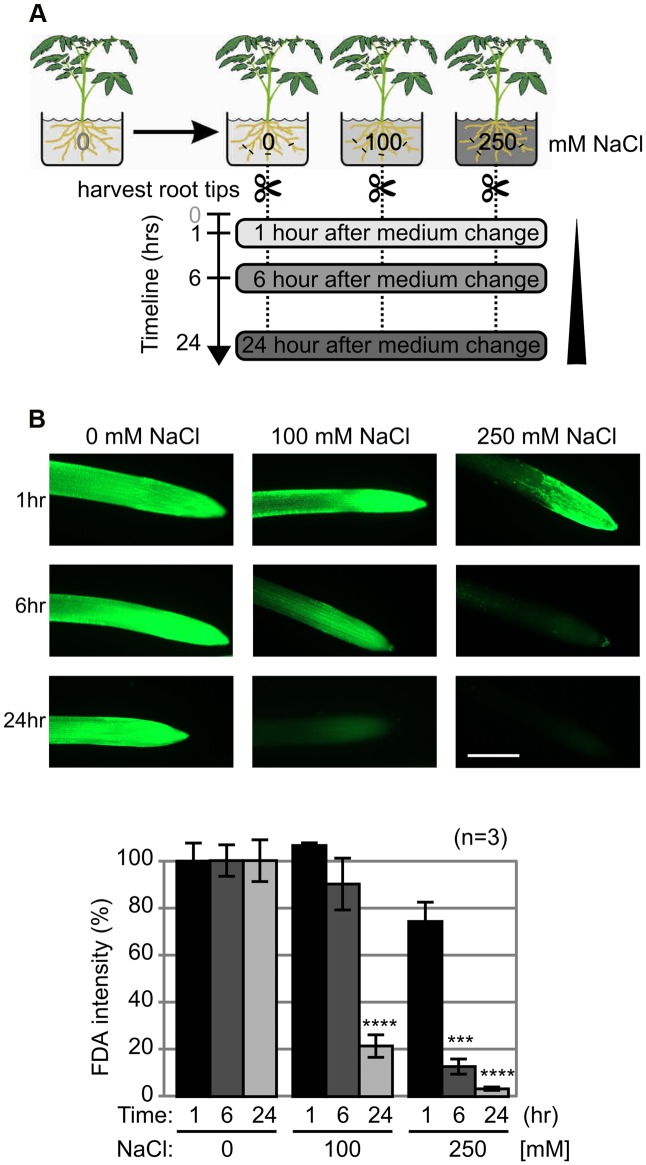
**Salt treatment induces loss of viability in tomato roots. (A)** Experimental assay. Tomato (*Solanum lycopersicum*) plants were grown in a hydroponic system and 5-weeks old plants were treated with 0-, 100- and 250 mM NaCl in the nutrient solution. Root tips were collected at 1, 6, and 24 h. **(B)** Loss of viability upon salt stress. Root tips were stained with fluorescein diacetate (FDA) to detect the viable cells. Top: representative images are shown. Scale bar, 0.5 mm. Bottom: fluorescent intensities of FDA fluorescence levels, when compared to the control. Error bars represent SEM of *n* = 3 biological replicates.

Salt stress is common in plants and is associated with the release of ROS. Higher salt concentration also triggers PCD (Hasegawa et al., 2000; Poór et al., 2014). To investigate salt stress in tomato roots, plants were treated with sublethal- (100 mM) and lethal (250 mM) concentrations of NaCl (**Figure 2A**). We studied the early stages of abiotic stress by collecting samples at 1, 6, and 24 h after salt exposure. Root tips were stained with FDA to detect and quantify viable cells. Low FDA staining after 6 h upon treatment with 250 mM NaCl indicates a massive and quick PCD that completes within 24 h (**Figure 2B**). By contrast, treatment with 100 mM NaCl caused a slower loss of viability, where decreased viability was detected only at 24 h.

### MV151 Labeling Uncovers Differential Proteasome Activity

**FIGURE 3:**
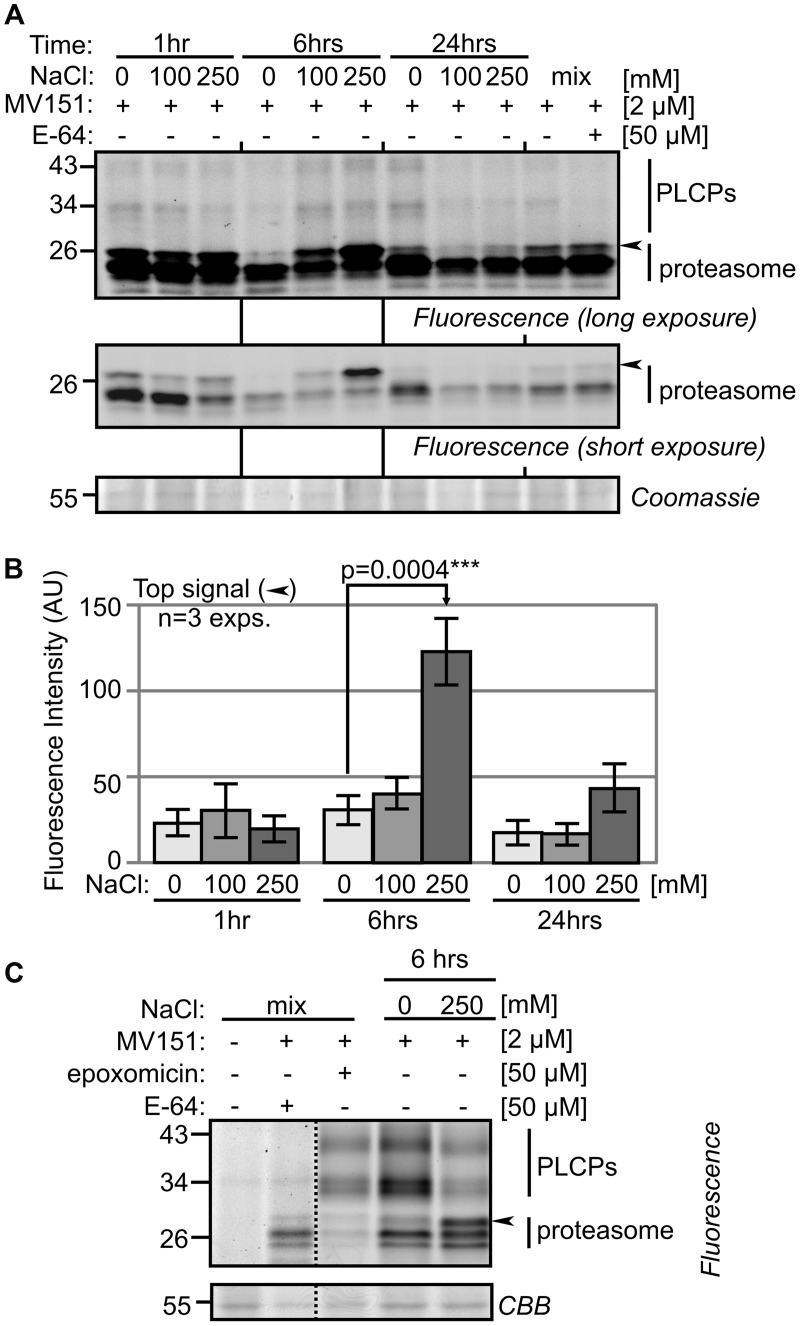
**MV151 activity profile changes upon salt stress in roots. (A)** Differential activity profiles with MV151. Tomato roots were treated with 0-, 100-, 250 mM NaCl. Root extracts were generated after 1-, 6- and 24 h and labeled with 2 μM MV151 at pH 6.0. A mix of all nine samples was pre-incubated with or without 50 μM E-64 and labeled with 2 μM MV151. Shown is a representative gel at long and short fluorescence exposure and upon coomassie staining. The other two experimental replicates are shown as Supplementary Figure S2. **(B)** Quantification of the upper differential MV151 signal (arrowhead) taken from three experimental replicates **(A)** (Supplementary Figure S2). Error bars represent SEM of *n* = 3 experimental replicates. **(C)** Differential signal is suppressed by proteasome inhibitor. The 6 h 0- and 250 mM NaCl treated samples were labeled with 2 μM MV151. A mix of the two samples was pre-incubated with or without 50 μM E-64 or epoxomicin and labeled with or without 2 μM MV151.

Papain-like cysteine Proteases and the proteasome have been implicated in stress and PCD. To examine the activity of both PLCPs and the proteasome, we first tested MV151, which labels both the proteasome and a subset of the PLCPs (Gu et al., 2010). MV151 labeling causes weak signals at 30–40 kDa which represent PLCPs and three stronger signals at 26 kDa that may represent the active proteasome subunits or PLCPs (**Figure 3A**). Interestingly, we detected a strongly activated band at ∼26 kDa with threefold higher intensity upon 250 mM salt treatment at 6 h (**Figure 3A**). This extra signal was robustly detected in all biological replicates (Supplementary Figure S2), and statistically significant upon quantification of fluorescence intensities from the three biological replicates (**Figure 3B**). To determine if this signal is caused by the proteasome or PLCPs, a competition assay was performed using proteasome inhibitor epoxomicin and PLCP inhibitor E-64. Pre-incubation with E-64 suppresses labeling at 30–40 kDa (**Figure 3C**), confirming that these signals are caused by PLCPs. The 26 kDa signals are not suppressed by E-64 (**Figure 3C**), indicating that these signals might be from the proteasome. Indeed, pre-incubation with the selective proteasome inhibitor epoxomicin suppresses labeling of all 26 kDa signals (**Figure 3C**), indicating that the significantly activated band is caused by the proteasome. Thus, although these MV151 labeling experiments did not display differential activities of PLCPs, it did uncover differential proteasome activity profiles.

### Salt Stress Alters the Activity Profile of Proteasome Catalytic Subunits

**FIGURE 4:**
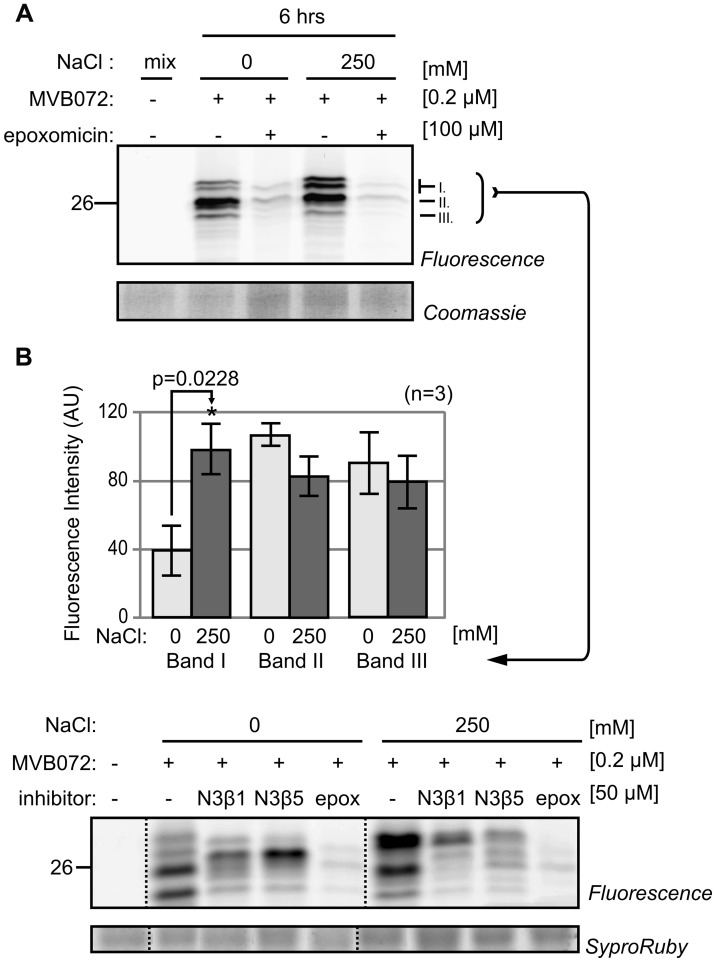
**Proteasome activity profile changes upon salt treatment. (A)** Tomato roots were treated with 0- and 250 mM NaCl and root extracts were generated after 6 h and pre-incubated with or without 100 μM epoxomicin and labeled with or without 0.2 μM MVB072. **(B)** Quantification of bands I–III indicated in **(A)**. Error bars represent SEM of *n* = 3 experimental replicates.

To confirm differential proteasome activity, we used a newly re-synthesized epoxomicin-based MVB072, which carries both a bodipy tag for fluorescent detection and a biotin tag for affinity purification (Kolodziejek et al., 2011). When compared to MV151, MVB072 is a much more selective proteasome probe without any known off targets, ideal to confirm differential proteasome activity in our samples (Kolodziejek et al., 2011). Importantly, MVB072 labeling displays the same altered activity profile upon salt treatment as MV151 labeling (**Figure 4A**). Quantification of fluorescence intensities of the various signals demonstrate a highly reproducible increased intensity of the upper signal at 6 h upon 250 mM NaCl treatment over multiple biological replicates, whilst other signals seem to reduce (**Figure 4B**).

### β2- and β5 Catalytic Subunits Migrate at Different Molecular Weight (MW) upon Lethal Salt Stress

**FIGURE 5:**
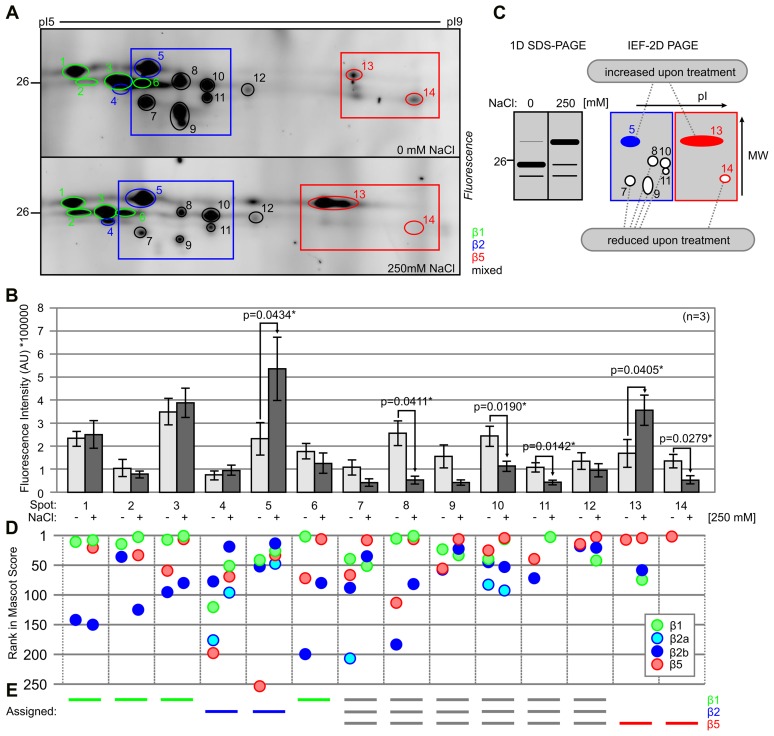
**Two-dimensional gels show molecular weight (MW) and pI shifts for labeled catalytic proteasome subunits. (A)** Tomato roots were treated with 0- and 250 mM NaCl and root extracts were generated after 6 h and labeled with 0.2 μM MVB072. Samples were separated on IEF 2D gel. Spots are highlighted with different colors: β1 (green); β2 (blue); and β5 (red). Framed sections focus on β2 (blue) and β5 (red) catalytic subunits. **(B)** Quantification of the fluorescence signals of **(A)**. Error bars represent SEM of *n* = 3 experimental replicates. **(C)** Schematic figures of 1D and 2D gel illustrating the effect of high salinity on the intensity of signals of β2 and β5 catalytic subunits. Closed and open spots indicate up- and down-regulated signals, respectively. **(D)** Ranking of detected catalytic subunits based on Mascot protein scores. **(E)** Assignment of catalytic subunits to some of the fluorescent spots, based on the detected proteins and their scores and ranking.

To identify the differentially active catalytic subunits of the proteasome, we separated MVB072-labeled proteomes of roots treated with and without 250 mM NaCl for 6 h by IEF and SDS gel electrophoresis. Over 14 fluorescent spots were robustly detected on 2D gels (**Figure 5A**). These fluorescent spots do not correlate with abundant proteins detected upon SYPRO Ruby staining of these gels (Supplementary Figure S3). The fluorescence intensity of each of these 14 spots was quantified using ImageJ and plotted in a histogram over three biological replicates (**Figure 5B**; Supplementary Figure S4). We detect significant increase of the fluorescence intensities of spots #5 and #13 and a significant decrease of fluorescence intensities of spots #8, #10, #11, #13, and #14, whilst fluorescence intensities of spots #7 and #9 were strongly increased upon salt treatment (**Figure 5B**). These changes in fluorescence intensities occurs in two regions in the 2D gel (boxed in **Figure 5A**) and correlates with the shift in fluorescent intensity on 1D gels, as illustrated in **Figure 5C**. At the acidic pI range, five spots (#7–11) showed reduced intensity upon salt stress, whilst one spot (#5) has increased signal intensity (blue box in **Figure 5C**). At basic pI range, the bottom signal (#14) decreases whilst the top signal (#13) intensifies upon salt stress (red box in **Figure 5C**).

A total of 28 fluorescent spots were excised from both gels and analyzed by MS. Twenty-seven of these spots contain catalytic subunits of the proteasome (Supplementary Figures S4–S6 and **Table S1**). In total, four different catalytic subunits were detected: Solyc07g016200.2.1 (β1), Solyc04g024420.2.1 (β2a), Solyc05g013820.2.1 (β2b), and Solyc05g056160.2.1 (β5), each with multiple unique peptides and significant Mascot protein scores (Supplemental Figures S5–S6 and Table S2).

β2a was identified in spots #5, #7, and #10 in the control treatment and in spots #4, #5, and #10 in the salt-treated sample (**Figure 5D**; Supplementary Figure S6). However, the majority of the MS signal in these spots comes from β2b (Supplementary Figure S6). This indicates that β2a is part of the proteasome, but contributes only a minor fraction, irrespective of the stress condition. These data do not support the hypothesis that the stress proteasome has a different β2a/β2b ratio.

To assign the fluorescence signal to a particular catalytic subunit, we ranked the protein scores in Mascot for each spot and highlighted the four detected catalytic subunits. This analysis shows that we often identified more than one catalytic subunit from several spots (**Figure 5D**). The identification of multiple subunits per spot might be caused by incomplete separation during IEF or by contamination during gel excision. We assigned the signal to a single catalytic subunit in case a single subunit ranks consistently high in a spot. This way we assigned β1 to spots #1, #2, #3, and #6; β2 to #4 and #5; β5 to #13 and #14, and we found stronger signals for multiple subunits in the remaining spots (#8–12; **Figure 5E**; Supplementary Figure S6). Taken together, these data indicate that salt stress induces a shift to a higher apparent MW for both labeled β2 and β5, and a shift to lower pI for labeled β5.

### Subunit-Selective Probes Confirm Differential β5 Activity Profile

**FIGURE 6:**
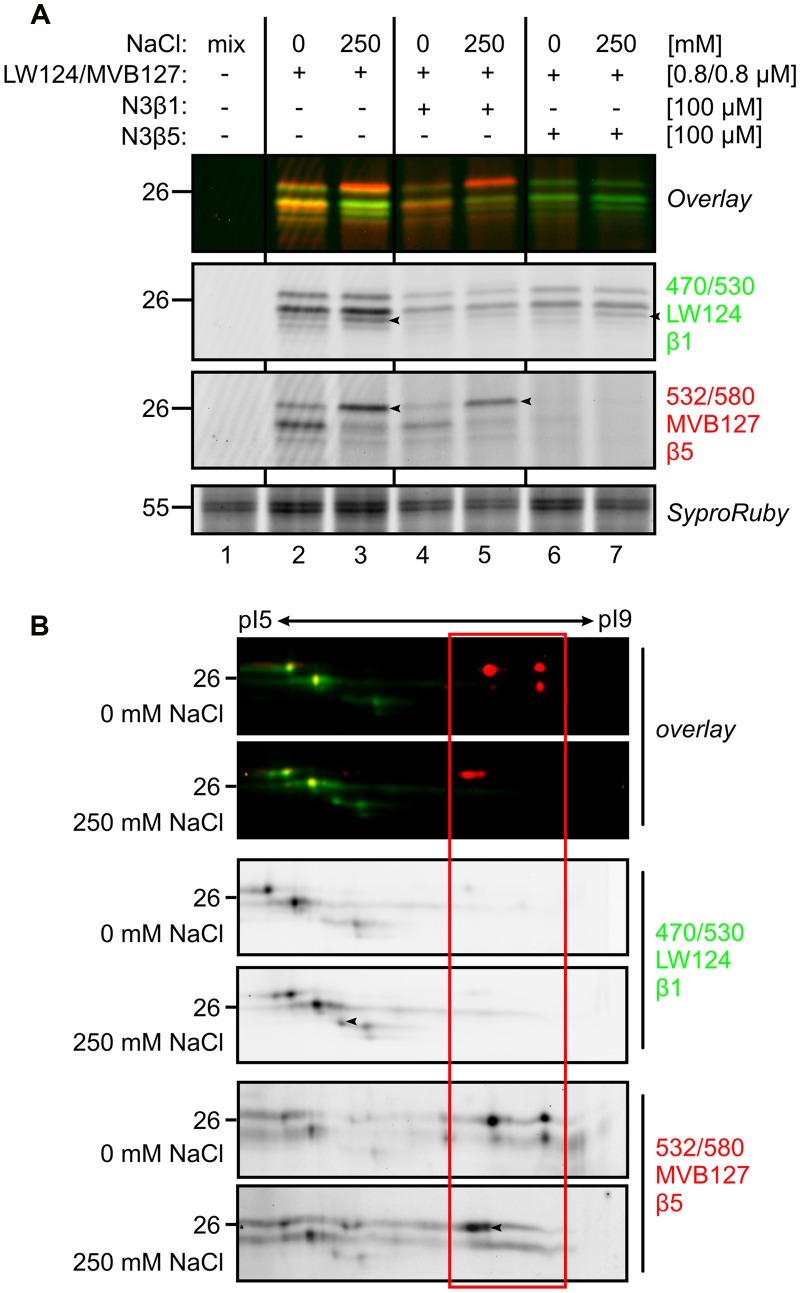
**Subunit-specific labeling confirms modification of β5. (A)** Tomato roots were treated with 0- and 250 mM NaCl and root extracts were generated after 6 h and pre-incubated with or without subunit-specific inhibitors, N3β1 and N3β5 and co- labeled with or without subunit-selective probes, LW124 (β1) and MVB127 (β5). Samples were separated on 1D gel. Differential signals are indicated by black arrowheads. **(B)** Tomato roots were treated with 0- and 250 mM NaCl and root extracts were generated after 6 h and co-labeled with LW124 (β1) and MVB127 (β5) and separated on IEF 2D gel. Differential signals are indicated by black arrowheads.

To confirm the identity of the shifting of the labeled catalytic subunits, the root extract was labeled with subunit-specific activity-based probes LW124 and MVB127 (Li et al., 2013; Misas-Villamil et al., 2017). LW124 is specific for β1 and has an epoxyketone reactive group and a fluorophore with excitation and emission wavelength at 470 and 530 nm, respectively. By contrast, MVB127 is selective for β5, carries a vinyl-sulfone reactive group and a fluorophore with excitation and emission wavelength at 532 and 580 nm, respectively. As they have different specifies and their excitation and emission wavelength are different, LW124 and MVB127 can be used by co-labeling (Misas-Villamil et al., 2017). Consistent with our earlier observations LW124 labeling does not display significant differences upon salt treatment, with the exception of on extra signal (arrowhead in **Figure 6A**). MVB127 labeling, however, shows a shift upward, confirming the MW shift of β5 (**Figure 6A**).

To confirm the composition of the LW124- or MVB127-labeled signals, we pre-incubated the samples with selective inhibitors, N3β1 and N3β5, which target β1 and β5, respectively (Misas-Villamil et al., 2017). N3β1 strongly suppressed labeling of LW124 (β1), whilst N3β5 blocked labeling of MVB127 (β5), respectively, verifying that the top differential MV151- and MVB072 signals contains a β5 subunit (**Figure 6A**). However, to a lesser extent, N3β1 also suppresses β5 labeling and N3β5 suppresses β1 labeling (**Figure 6A**), which can be explained by some cross reactivity of the inhibitors, but also by allosteric effects caused by subunit inhibition, including sterical hindrance in the proteolytic chamber.

To confirm the specific labeling and the pI/MW shifts of the catalytic subunits, we analyzed LW124/MVB127 co-labeled samples on 2D IEF-PAGE gels. The labeling pattern confirms the MS data: spots in the acidic range are labeled with LW124, consistent with being β1-derived signals, whereas the spots in the basic pI are labeled with MVB127, consistent with being β5-derived (**Figure 6B**). We do not detect cross reactivity of LW124 on β5, but there is some cross reactivity of MVB127 on β1 (**Figure 6B**). Consistent with proceeding data, the MVB127-labeled signals shift increase in MW and shift to more acidic pI upon salt treatment, even though in this case we detect some of this ‘stress β5 isoform’ also in plants treated without salt (**Figure 6B**). By contrast, LW124-labeled signals are constant in the samples treated with or without salt, with the exception of one additional signal that correlates to the signal detected on 1D gels (arrowhead in **Figures 6A,B**). This confirms that most of labeled β1 remains unaltered upon salt stress. Taken together, these data confirm a pI/MW shift for labeled β5 upon salt treatment.

### Molecular Weight Shift of Labeled β2 or β5 Is Not Affected by Phosphatase or Glycosidase Treatments

**FIGURE 7:**
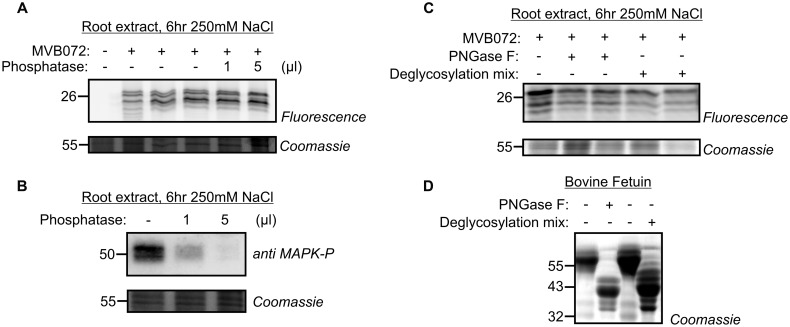
**Phosphatase and glycosidase treatments do not affect altered proteasome activity profile. (A)** Root extracts of 250 mM treated samples, labeled by MVB072 were treated with alkaline phosphatase at different conditions. **(B)** Dephosphorylation of MAP kinase, used as a positive control, detected by an anti-phosphoMAPK antibody. **(C)** Root extracts of 250 mM treated-samples, labeled by MVB072 were treated with and without PNGase F or deglycosylation mix. **(D)** Enzymatic deglycosylation of Bovine Fetuin was used as a positive control.

Because of the shifts of denatured labeled β2 and β5 subunits when separated on 1D and 2D gels, we hypothesize that the MW is caused by a PTM of the labeled β2 and β5 subunits. Phosphorylation and glycosylation are two PTMs described for the proteasome (Bose et al., 2004; Zong et al., 2008; Scruggs et al., 2012). To test if the MW shifts are caused by phosphorylation, we treated the labeled samples with alkaline phosphatase. However, treatment of MVB072-labeled sample with alkaline phosphatase did not affect the shifted signal (**Figure 7A**). Probing the same extract with an antibody against phosphorylated MAP kinases showed a strong reduction, indicating that protein dephosphorylation worked in this assay (**Figure 7B**).

To test if the MW shifts are caused by differential glycosylation, we incubated the MVB072-labeled sample with PNGase F to remove *N*-glycans or with deglycosylation mix to remove both *N*- and *O*-glycans using a mixture of PNGase F, *O*-Glycosidase, Neuraminidase, β1–4 Galactosidase, en β*-N*-Acetylglucosaminidase. Neither of these treatments affected the labeling profile, suggesting that the MW shift is not caused by *N*- or *O*-glycosylation (**Figure 7C**). By contrast, Bovine Fetuin, a glycoprotein standard used as a positive control, did shift upon both treatments, indicating that deglycosylation worked in this assay (**Figure 7D**).

## Discussion

We discovered robust alteration in the activity profile of proteasome catalytic subunits during salt stress. Both labeled catalytic β2 and β5 subunits shifted to a higher MW and β5 also has a negative pI shift upon salt stress. The recurrence of a normal proteasome profile at 24 h upon treatment with 250 mM NaCl indicates that this change is reversible and occurs concomitantly with PCD. The reason for proteasome modification could be an altered preference for substrates. Salt stress induces protein oxidation (Mano et al., 2014) and an altered proteasome would be required to degrade these oxidized proteins. The three catalytic subunits have different peptidase activity and their modification may cause changes in their activity and specificity.

There are several molecular mechanisms that might underpin MW/pI shifts of catalytic subunits. In animals, standard proteasome subunits are replaced in nascent proteasome complexes by highly homologous β1i, β2i, and β5i subunits, which more efficiently produce antigenic peptides in response to infection (Tanaka and Kasahara, 1998) and more efficiently degrade oxidized proteins (Seifert et al., 2010). Replacements by the i-20S proteasome subunits open the central chamber to allow access of more proteins to the catalytic core (Groettrup et al., 2010). Also, the immunoproteasome has reduced caspase-like activity (Ferrington and Gregerson, 2012). The existence of an immunoproteasome in tobacco was strongly suggested by the stress-induced accumulation of transcripts encoding β1, α3, and α6 subunits (Suty et al., 2003). We can, however, exclude the mechanism of subunit replacement in tomato because tomato has single genes encoding six of the seven β subunits. Tomato carries two genes encoding substantially different β2 subunits, but our MS analysis did not suggest a changed subunit assembly upon salt stress. However, although different genes encoding the different β5 isoforms can be excluded, alternative splicing could still result in different β5 isoforms from the same gene. In animals, for instance, alternative splicing of transcripts encoding proteasome subunits can alter the activity and substrate specificity of 20S proteasome (Kawahara et al., 2000).

### Post-translational Regulation of the Proteasome

Post-translational modification is a likely molecular mechanism underpinning the shift in MW and pI of catalytic subunits. In animals, multiple PTMs have been described for proteasome subunits such as phosphorylation, glycosylation, ubiquitination, oxidation, glutathionylation, and nitrosylation (Bose et al., 2004; Ventadour et al., 2007; Zong et al., 2008; Scruggs et al., 2012; Cui et al., 2014). PTMs can regulate the stability of the proteasome, alter the assembly of different subunits and change the degradation pattern of proteasome or activity profile of catalytic subunits (Bose et al., 2004).

First, protein phosphorylation can cause both MW and pI changes (Zong et al., 2008). Phosphorylation of a serine residue in the α6 subunit has been studied in root tips of rice (Umeda et al., 1997). Phosphorylation also likely causes a mass increment of β1din during the induction of defense in tobacco (Suty et al., 2003). There are several predicted phosphorylation sites in catalytic subunits. The β2 subunit contains eight Ser, five Thr, four Tyr residues, whereas the β5 subunit contains 10 Ser, one Thr, and three Tyr residues (Supplementary Figure S3). However, searches or our MS data allowing phosphate modifications, did not reveal any phosphorylated peptides from the β2 or β5 subunits. In addition, phosphatase treatment did not affect the MVB072 activity profile in tomato roots upon 250 mM NaCl treatment (**Figure 7A**) suggesting that phosphorylation is not the underlying mechanism of the altered proteasome upon salt stress. However, phosphorylation cannot be ruled out because some phosphorylations can endure alkaline phosphatase treatment.

Second, proteasome subunits can be regulated by glycosylation. For instance *O*-Glycosylation reversibly inhibits proteasome function via modification of Rpt2 ATPase in the 19S regulatory particle of the proteasome in animals (Zhang et al., 2003). Likewise, *N*-Glycosylation is a key PTM of specific proteins during osmotic stress adaptation in plants (Koiwa et al., 2003). However, deglycosylation by PNGase or deglycosidase mix had no effect on the MW shift of β5 (**Figure 7C**) indicating that also glycosylation is not the underlying mechanism.

Third, ubiquitination is common on proteasome subunits. Both β2 and β5 catalytic subunits are ubiquitinated at Lys residues (Ventadour et al., 2007; Kim et al., 2013). However, the predicted MW shift of mono- or polyubiquitinated β2 and β5 is too large (>8.5 kDa) to explain the observed ∼2 kDa MW shift in the altered proteasome. The same argument excludes sumoylation as a PTM underlying the observed shifts in MW.

In addition, ROS production leads to accumulation of oxidatively modified proteins (e.g., carbonyl compounds) that alter the function of enzymes (Basset et al., 2002). There is evidence of carbonylation of the 20S proteasome in response to carbon starvation in maize root tips (Basset et al., 2002). Furthermore, glutathionylation has been detected on each of the β5 subunits of the plant proteasome (Dixon et al., 2005). β2 has four and β5 has two *Cys* residues which may explain a 2 kDa upon *S*-glutathionylation. Finally, *Cys* residues can also be modified by reactive nitrogen species, which are released during stress (Hess et al., 2005). However, the use of reducing agents during our sample preparation would probably remove oxidation, glutathionylation, and nitrosylation from *Cys* residues, so these PTMs are not likely to explain the MW/pI shifts that we detected. However, many additional PTMs are known and they can also be combined in several ways. Also a combination of PTMs might result in the observed MW/pI shifts in the activity profile of β2 and β5 catalytic subunits.

## Conclusion

We discovered an altered proteasome activity profile at the early stage of salt stress-induced PCD in tomato roots. Modification of proteasome profile is probably caused by yet unidentified covalent modification of β2 and β5 proteasome catalytic subunits, which is not caused by differential subunit assembly, and may not be caused by phosphorylation, glycosylation or ubiquitination of catalytic subunits. This modification of the proteasome catalytic subunits is reversible and correlates with the need to degrade oxidized proteins during biotic and abiotic stress. Further work can now focus at the structural and functional elucidation of the stress-induced proteasome to determine its role in stress responses in plants.

## Data Availability

The mass spectrometry proteomics data have been deposited to the ProteomeXchange Consortium via the PRIDE (Vizcaíno et al., 2016) partner repository (https://www.ebi.ac.uk/pride/archive/) with the dataset identifier PXD005266. The samples have been renamed as summarized in Supplementary Table S3.

## Author Contributions

JK, BC, TH, and RvdH designed experiments. JK performed most experiments. PP performed microscopy. FK performed mass spectrometry. JK, FK, JM-V, and RvdH analyzed the data. BX, MK, and HO contributed materials. JK and RvdH wrote the manuscript with suggestions from all coauthors. IT and RvdH supervised the project.

## Conflict of Interest Statement

The authors declare that the research was conducted in the absence of any commercial or financial relationships that could be construed as a potential conflict of interest.

## Footnotes

1 www.solgenomics.net

2 swissmodel.expasy.org

3 http://www.gelifesciences.com

4 solgenomics.net

5 www.solgenomics.org

## Supplementary Material

TABLE S1 **Peptides and proteins of tomato detected by MS in 2D gels**.

## Abbreviations

ABPP: activity-based protein profiling
IEF: isoelectric focusing
PCD: programmed cell death
PLCP: papain-like Cys protease
PTM: post-translational modification
ROS: reactive oxygen species
