# Supplementary material for: Proteasome Activity Profiling Uncovers Alteration of Catalytic β2 and β5 Subunits of the Stress-Induced Proteasome during Salinity Stress in Tomato Roots
Source: Front Plant Sci. 2017 Feb 3;8:107. doi: 10.3389/fpls.2017.00107 (PMC5289967; doi:10.3389/fpls.2017.00107)
Supplement: Supplementary file 2 [file Data_Sheet_1.pdf]

SUPPLEMENTAL FIGURES S1-S6 and TABLES S2-S3

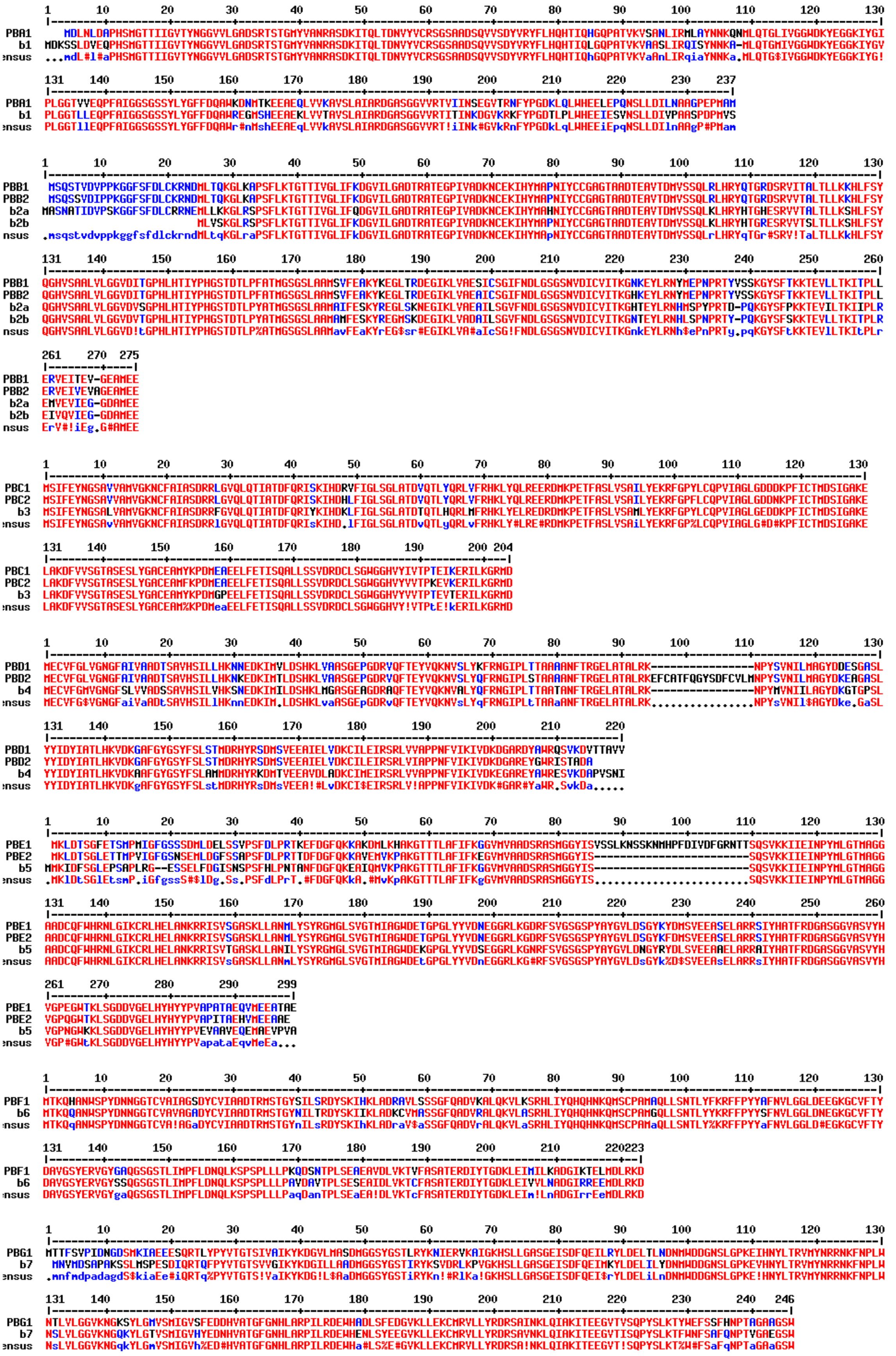

Figure S1 Alignment of Arabidopsis and tomato β subunit protein sequences per subfamily

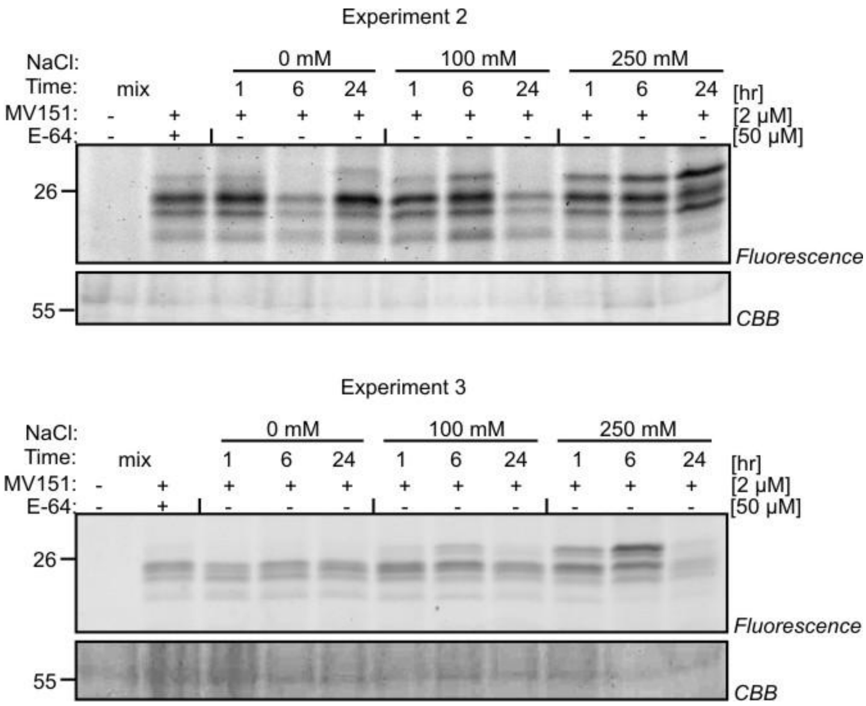

Figure S2 Two additional experimental replicates of MV151 labeling.

Tomato roots were treated with 0-, 100- and 250 mM NaCl and root extracts were generated after 1, 6 and 24 hours and labeled with 2 µM MV151. A mix of all nine samples was pre-incubated with or without 50 µM E-64 and labeled with 2 µM MV151.

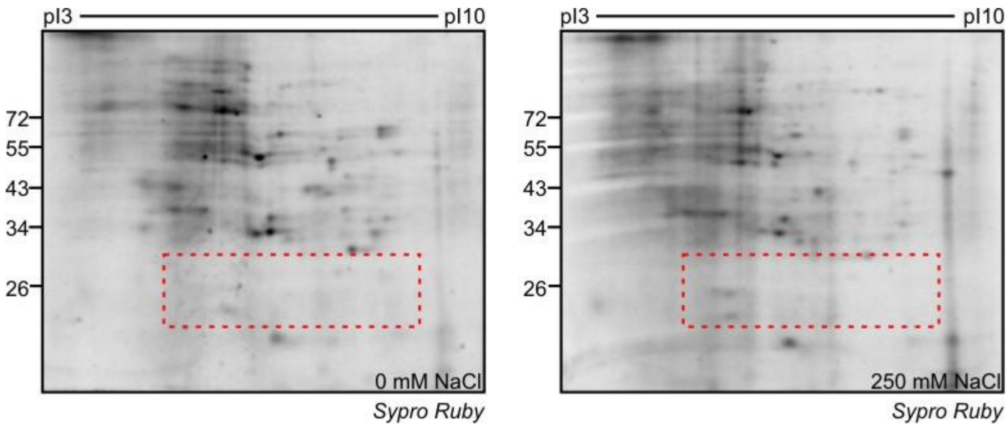

Figure S3: Sypro Ruby-stained 2D gels, shown in Figure 5A. The regions that are shown in Figure 5A are highlighted with the red box.

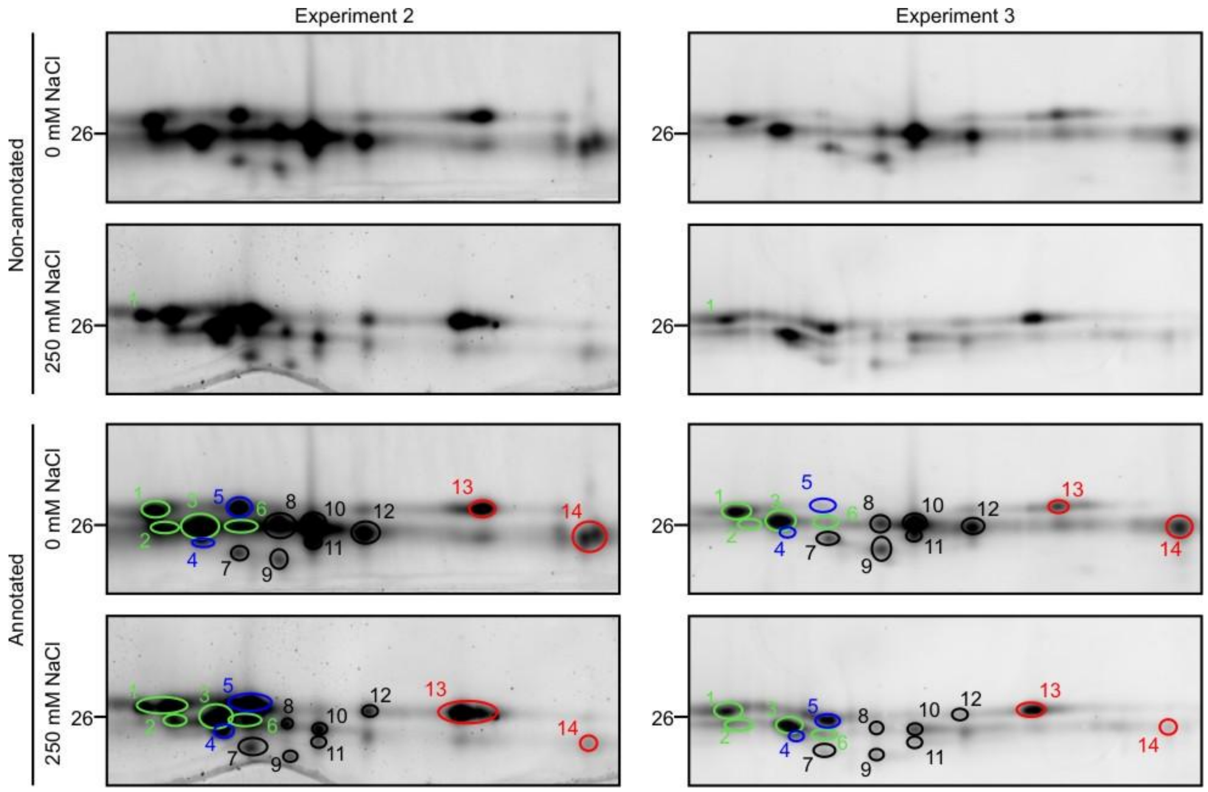

Figure S4 Two additional biological replicates of fluorescent 2D gels.

Tomato roots were treated with 0- and 250 mM NaCl and root extracts were generated after 6 hours and labeled with 0.2 µM MVB072. Samples were separated on IEF 2D gel. Spots are highlighted with different colors: β1 (green); β2 (blue); and β5 (red).

>Solyc07g016200.2.1 β1

MDKSSLDVEQPHSMGTTIIGVTYNGGVVLGADSRTSTGMYVANRASDKITQLTDNVYVCRSGSAADSQ
VVSDYVRYFLHQHTIQLGQPATVKVAASLIRQISYNNKAMLQTGMIVGGWDKYEGGKIYGVPLGGTLL
EQPFAIGGSGSSYLYGFFDQAWREGMSHEEAEKLVVTAVSLAIARDGASGGVVRTITINKDGVKRKFY
PGDTLPLWHEEIESVNSLLDIVPAASPDPMVS

>Solyc04g024420.2.1 β2a

MASNATIDVPSKGGFSFDLCRRNEMLLKKGLRSPSFLKTGTTIVGLIFQDGVILGADTRATEGPIVAD
KNCEKIHYMAHNIYCCGAGTAADTEAVTDMVSSQLKLHRYHTGHESRVVTALTLLKSHLFSYQGHVSA
ALVLGGVDVSGPHLHTIYPHGSTDTLPYATMGSGSLAAMAIFESKYREGLSKNEGIKLVAEAILSGVF
NDLGSGSNVDICVITKGHTEYLRNHMSPYPRTDPQKGYSFPKKTEVILTKIIPLREMVEVIEGGDAME
E

>Solyc05g013820.2.1 β2b

MLVSKGLRSPSFLKTGTTIVGLIFKDGVILGADTRATEGPIVADKNCEKIHYMAPNIYCCGAGTAADT
EAVTDMVSSQLKLHRYHTGRESRVVTSLTLLKSHLFSYQGHVSAALVLGGVDVTGPHLHTIYPHGSTD
TLPYATMGSGSLAAMAMFESKYREGMSKDEGIKLVADAILSGVFNDLGSGSNVDICVITKGNTEYLRN
HLSPNPRTYPQKGYSFSKKTEVLLTKITPLREIVQVIEGGDAMEE

>Solyc05g056160.2.1 β5

MMKIDFSGLEPSAPLRGESSELFDGISNSPSFHLPNTANFDGFQKEAIQMVKPAKGTTTLAFIFKGGV
MVAADSRASMGGYISSQSVKKIIEINPYMLGTMAGGAADCQFWHRNLGIKCRLHELANKRRISVTGAS
KLLANILYSYRGMGLSVGTMIAGWDEKGPGLYYVDSEGGRLKGNRFSVGSGSPYAYGVLDNGYRYDLS
VEEAAELARRAIYHATFRDGASGGVASVYHVGPNGWKKLSGDDVGELHYHYYPVEVAAVEQEMAEVPV
A

unique and ambiguous peptides

Figure S5 Position of identified peptides on proteasome sequences. Unique peptides are highlighted in red, ambiguous peptides are highlighted in yellow, peptides that overlap with a larger peptide are underlined. Catalytic Thr is highlighted in blue. The prodomain is printed in grey.

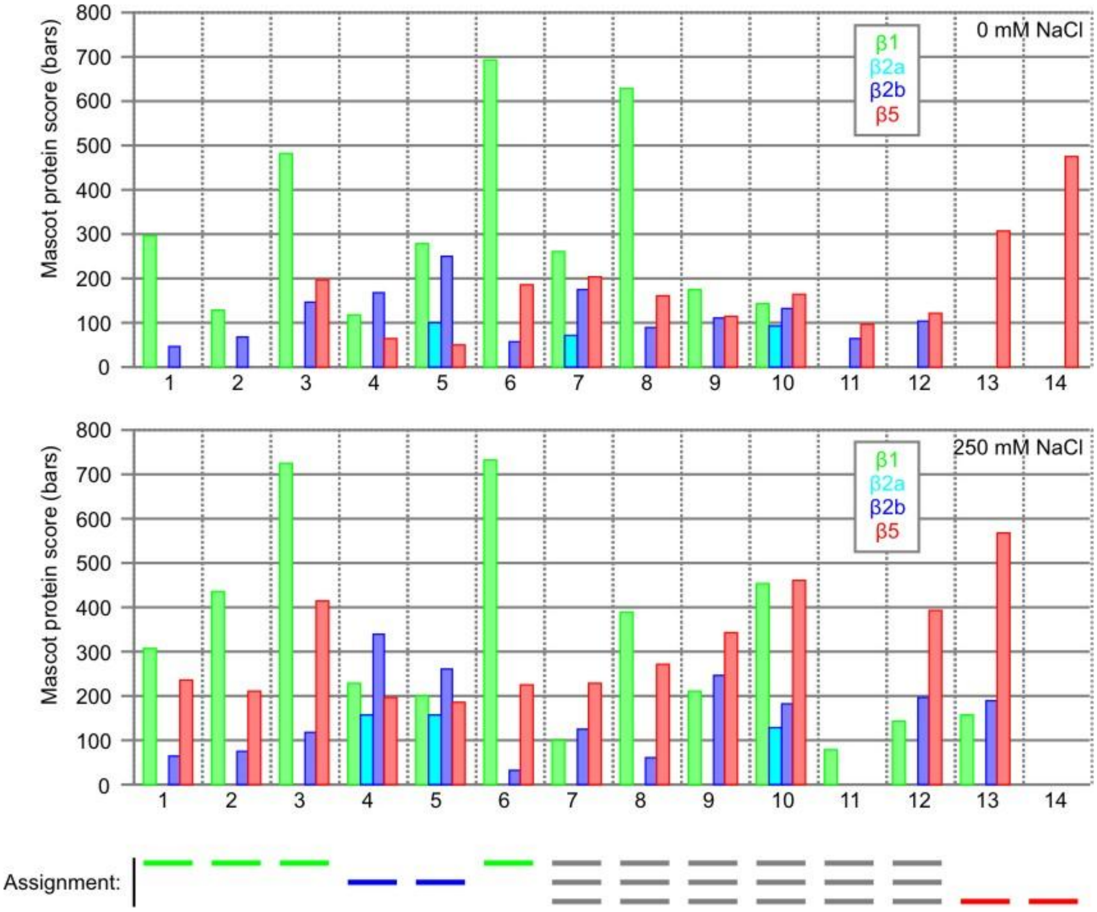

Figure S6 Mascot scores of identified proteasome catalytic subunits after separation with IEF 2D PAGE. Mascot scores are show for each of the four detected catalytic subunits. The assigned subunit composition is shown on the bottom.

Table S2 Identified peptides of catalytic subunits.

Peptide sequence	u/a	PSM	Subunit	Accesion
ITQLTDNVYVCR	u	29	β1	Solyc07g016200.2.1
LVVTAVSLAIAR	u	32	β1	Solyc07g016200.2.1
AM*LQTGM*IVGGWDK	u	8	β1	Solyc07g016200.2.1
DGASGGVVR	u	19	β1	Solyc07g016200.2.1
VAASLIR	u	21	β1	Solyc07g016200.2.1
TSTGM*YVANR	u	18	β1	Solyc07g016200.2.1
EGMSHEEAEK	u	5	β1	Solyc07g016200.2.1
SGSAADSQVVSDYVR	u	20	β1	Solyc07g016200.2.1
ASDKITQLTDNVYVCR	u	12	β1	Solyc07g016200.2.1
AM*LQTGM*IVGGWDKYEGGK	u	17	β1	Solyc07g016200.2.1
TITINKDGVK	u	7	β1	Solyc07g016200.2.1
YFLHQHTIQLGQPATVK	u	9	β1	Solyc07g016200.2.1
TSTGMYVANR	u	2	β1	Solyc07g016200.2.1
AM*LQTGMIVGGWDK	u	3	β1	Solyc07g016200.2.1
AMLQTGMIVGGWDK	u	1	β1	Solyc07g016200.2.1
QISYNNK	u	1	β1	Solyc07g016200.2.1
ATEGPIVADK	a	16	β2a	Solyc04g024420.2.1
ATEGPIVADKNCEK	a	6	β2a	Solyc04g024420.2.1
TEVILTK	a	5	β2a	Solyc04g024420.2.1
KTEVILTK	a	10	β2a	Solyc04g024420.2.1
VVTALTLLK	u	4	β2a	Solyc04g024420.2.1
EM*VEVIEGGDAMEE	u	1	β2a	Solyc04g024420.2.1
EMVEVIEGGDAMEE	u	1	β2a	Solyc04g024420.2.1
DGVILGADTR	a	3	β2b	Solyc05g013820.2.1
VVTSLTLLK	u	18	β2b	Solyc05g013820.2.1
ATEGPIVADK	a	16	β2b	Solyc05g013820.2.1
EIVQVIEGGDAM*EE	u	11	β2b	Solyc05g013820.2.1
GNTEYLR	u	20	β2b	Solyc05g013820.2.1
KTEVLLTK	a	21	β2b	Solyc05g013820.2.1
NHLSPNPR	u	1	β2b	Solyc05g013820.2.1
EIVQVIEGGDAMEE	u	9	β2b	Solyc05g013820.2.1
ATEGPIVADKNCEK	a	6	β2b	Solyc05g013820.2.1

TEVLLTK	a	6	β2b	Solyc05g013820.2.1
YDLSVEEAAELAR	u	30	β5	Solyc05g056160.2.1
GPGLYYVDSEGGR	u	21	β5	Solyc05g056160.2.1
LLANILYSYR	u	22	β5	Solyc05g056160.2.1
GGVMVAADSR	u	15	β5	Solyc05g056160.2.1
GGVM*VAADSR	u	21	β5	Solyc05g056160.2.1
ASMGGYISSQSVK	u	13	β5	Solyc05g056160.2.1
ASM*GGYISSQSVK	u	12	β5	Solyc05g056160.2.1
GM*GLSVGTM*IAGWDEK	u	5	β5	Solyc05g056160.2.1
GMGLSVGTM*IAGWDEK	u	5	β5	Solyc05g056160.2.1
FSVGSGSPYAYGVLDNGYR	u	6	β5	Solyc05g056160.2.1
GMGLSVGTMIAGWDEK	u	1	β5	Solyc05g056160.2.1

(u) unique peptide; (a) ambiguous peptide; (PSM) peptide spectral count; M*, oxidized methionine

These data are a summary of all the detected peptides from all the analyzed spots taken from both 2D gels, extracted from MaxQuant.

Table S3 Renamed samples from 2D gels

0 mM NaCl		250mM NaCl	
Fig4A	Database	Fig4A	Database
1	ACE_0037_JK01	1	ACE_0037_JK15
2	ACE_0037_JK02	2	ACE_0037_JK16
3	ACE_0037_JK03	3	ACE_0037_JK17
4	ACE_0037_JK04	4	ACE_0037_JK18
5	ACE_0037_JK05	5	ACE_0037_JK19
6	ACE_0037_JK06	6	ACE_0037_JK20
7	ACE_0037_JK07	7	ACE_0037_JK21
8	ACE_0037_JK08	8	ACE_0037_JK22
9	ACE_0037_JK09	9	ACE_0037_JK23
10	ACE_0037_JK10	10	ACE_0037_JK24
11	ACE_0037_JK11	11	ACE_0037_JK25
12	ACE_0037_JK12	12	ACE_0037_JK26
13	ACE_0037_JK13	13	ACE_0037_JK27
14	ACE_0037_JK14	14	ACE_0037_JK28
